# A Clinically-Compatible Workflow for Computer-Aided Assessment of Brain Disease Activity in Multiple Sclerosis Patients

**DOI:** 10.3389/fmed.2021.740248

**Published:** 2021-11-03

**Authors:** Benoit Combès, Anne Kerbrat, Guillaume Pasquier, Olivier Commowick, Brandon Le Bon, Francesca Galassi, Philippe L'Hostis, Nora El Graoui, Raphael Chouteau, Emmanuel Cordonnier, Gilles Edan, Jean-Christophe Ferré

**Affiliations:** ^1^Univ Rennes, Inria, CNRS, Inserm IRISA UMR 6074, Empenn ERL U 1228, Rennes, France; ^2^Neurology Department, Rennes University Hospital, Rennes, France; ^3^IRT bcom, Rennes, France; ^4^Biotrial, Rennes, France; ^5^CHU Rennes, Department of Neuroradiology, Rennes, France

**Keywords:** computer aided diagnosis, radiology, lesion activity, MRI, Multiple Sclerosis

## Abstract

Over the last 10 years, the number of approved disease modifying drugs acting on the focal inflammatory process in Multiple Sclerosis (MS) has increased from 3 to 10. This wide choice offers the opportunity of a personalized medicine with the objective of no clinical and radiological activity for each patient. This new paradigm requires the optimization of the detection of new FLAIR lesions on longitudinal MRI. In this paper, we describe a complete workflow—that we developed, implemented, deployed, and evaluated—to facilitate the monitoring of new FLAIR lesions on longitudinal MRI of MS patients. This workflow has been designed to be usable by both hospital and private neurologists and radiologists in France. It consists of three main components: (i) a software component that allows for automated and secured anonymization and transfer of MRI data from the clinical Picture Archive and Communication System (PACS) to a processing server (and vice-versa); (ii) a fully automated segmentation core that enables detection of focal longitudinal changes in patients from T1-weighted, T2-weighted and FLAIR brain MRI scans, and (iii) a dedicated web viewer that provides an intuitive visualization of new lesions to radiologists and neurologists. We first present these different components. Then, we evaluate the workflow on 54 pairs of longitudinal MRI scans that were analyzed by 3 experts (1 neuroradiologist, 1 radiologist, and 1 neurologist) with and without the proposed workflow. We show that our workflow provided a valuable aid to clinicians in detecting new MS lesions both in terms of accuracy (mean number of detected lesions per patient and per expert 1.8 without the workflow vs. 2.3 with the workflow, *p* = 5.10^−4^) and of time dedicated by the experts (mean time difference 2′45″, *p* = 10^−4^). This increase in the number of detected lesions has implications in the classification of MS patients as stable or active, even for the most experienced neuroradiologist (mean sensitivity was 0.74 without the workflow and 0.90 with the workflow, *p*-value for no difference = 0.003). It therefore has potential consequences on the therapeutic management of MS patients.

## Introduction

Magnetic Resonance Imaging (MRI) currently plays a central role in the diagnosis, prognosis and follow-up of patients with Multiple Sclerosis (MS) (1). In particular, the identification of new FLAIR hyperintense lesions between two longitudinal MRI scans allows: (i) to confirm the diagnosis of relapsing-remitting MS if the criterion of dissemination in time is not met on the first MRI scan (2); (ii) to provide information on the prognosis of the disease (3); (iii) to evaluate for each patient the current efficacy of its disease modifying treatment. Indeed, in recent years, the number of disease-modifying treatments for MS has increased significantly (1). In particular, highly effective second-line immunosuppressive treatments have become available and the number of first-line treatments has increased. However, these treatments are not without potential side-effects. The challenge is therefore to prescribe the right treatment to the right patient and to monitor its effectiveness closely. In this context, the concept of No Evidence of Disease Activity (NEDA) has emerged (4) and implies that MS patients have neither clinical relapse nor new FLAIR lesions on their follow-up MRI under treatment. An annual follow-up by brain MRI is therefore currently recommended, at least during the first year of treatment (5, 6), and the comparison of annual MRI scans is frequently performed by the radiologists and neurologists in charge of the follow-up of MS patients. However, this comparison is a complex and mentally demanding task that often leads to an underestimation of lesion accumulation, even for most experienced radiologists (7). Consequently, there is a need for dedicated systems that can provide clinicians, regardless of their level of expertise, an aid for accurate and robust detection of new FLAIR MS lesions. The ultimate goal of these systems will be to reduce the underestimation of patients wrongly reported as having no or few new lesions as well as the associated expert dependencies, resulting in better therapeutic decisions. For many years, different methods have been proposed to address this issue (8).

More recently, standardization of MR imaging acquisitions and data-transfer protocols as well as advances in computer vision methods have offered the premises for an end-to-end workflow for computer-aided comparative analysis of longitudinal MRI data. In particular, thanks to the development of deep learning techniques, powerful tools for the automatic segmentation of new MS lesions have been proposed in the context of academic research on the one hand [e.g., (7, 9, 10)], and integrated into commercial products on the other hand (11). However, the added-value of these tools in clinical practice is not well-documented, especially regarding therapeutic strategy and disability progression. In addition, the question of their integration into clinical practice is generally not addressed. Finally, commercially available solutions based on Artificial Intelligence often lack available scientific evidence in peer-reviewed Journals (11) and their high cost limits their deployment for patient care. Consequently, such tools have not yet been adopted in routine clinical practice by the majority of radiologists and neurologists.

Within this context, we launched the MUSIC project (an acronym for MUltiple Sclerosis Image Checkout) in 2017 in Brittany, a region in the north-west of France. The objective of this project was to develop, deploy and evaluate a fully-integrated clinical workflow allowing to improve detection of new brain lesions in MS patients. The system has been designed to be usable by both hospital and private radiologists and neurologists in Brittany. The MUSIC project also included centralized storage of MS patients' MRI data so that their data could be accessed and compared even if they moved from one center to another for their MRI or neurological follow-up. The first “proof of concept” phase of the project reported in this article was deployed in 5 centers (2 university hospitals, 2 local hospitals, 1 private radiology center).

The MUSIC workflow consists of three main components: (i) a software component that allows for automated and secured anonymization and transfer of MRI data from the clinical Picture Archive and Communication System (PACS) to a processing server (and vice versa); (ii) a fully automated MR image segmentation core that enables detection of new lesions from patients T1 weighted, T2 weighted and FLAIR brain acquisitions, and (iii) a dedicated web viewer that provides an intuitive visualization of new lesions to the clinical staff, easy to show to the patients. These elements allow clinicians to access and visualize enhanced patient data scanned in any connected clinical center, even without being linked to a clinical PACS. In the present paper, we first illustrate the MUSIC project workflow. Second, we assess the performance of three clinicians, with different levels of expertise, in identifying new MS lesions on follow-up MRI scans, with and without the proposed workflow. For this evaluation, we used longitudinal pairs of scans from 54 MS patients.

## Materials and Methods

**Figure 1:**
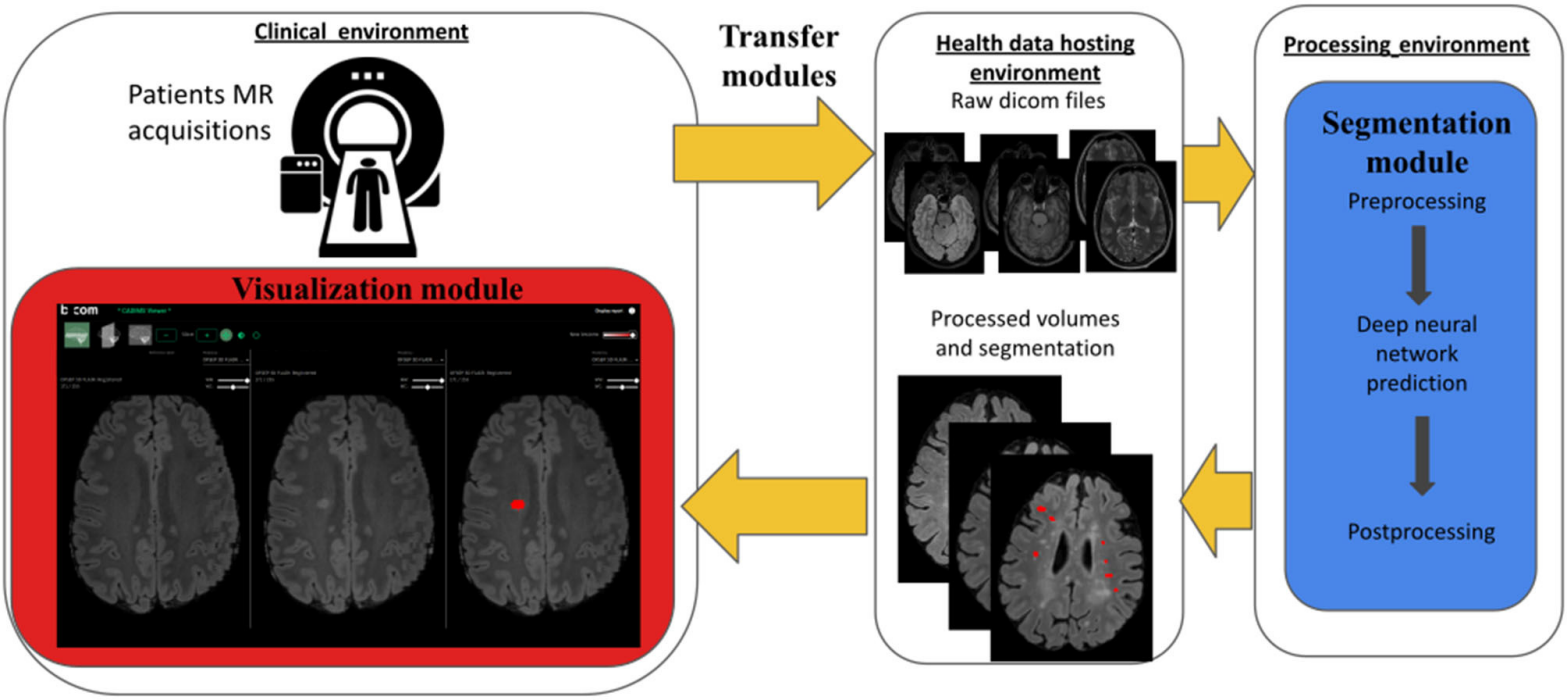
Workflow overview. Colored elements were specifically designed and developed in the context of our MUSIC project and consists of (i) a set of Transfer and Storage Modules (yellow), (ii) a Segmentation Module (blue) and (iii) a Visualization Module (red). Briefly, after being stored in the clinical PACS, MR images are pseudonymised and securely transferred into a processing hosting, where images are processed so that new lesions are automatically segmented. Then the resulting processed images and associated new lesions segmentation maps are returned to the clinical data hosting platform where they can be visualized in a dedicated web MRI viewer.

Figure 1 summarizes the overall MUSIC project workflow. Briefly, after being stored in the clinical local PACS, MR images are pseudonymized and securely transferred into a processing hosting, where images are processed and new lesions are automatically segmented using a deep neural network. Then, the processed images and corresponding segmentation maps are transferred back to the clinical hosting from which they can be efficiently visualized in a dedicated web MRI viewer. In the following, we describe the three main elements of the workflow: the transfer and storage modules (section The transfer and storage modules: Servers interoperability and data access), the segmentation module (section The segmentation module: Detection of new lesions from longitudinal brain MR images) and the visualization module (section The visualization module: Efficient and adapted reporting). Then, in section Evaluation of the MUSIC workflow, we present a set of experiments that we designed and carried out to evaluate the radiologist and the neurologist performances in identifying new FLAIR lesions between two sets of MRIs of MS patients with and without the workflow.

### Workflow Description

#### The Transfer and Storage Modules: Servers Interoperability and Data Access

**Figure 2:**
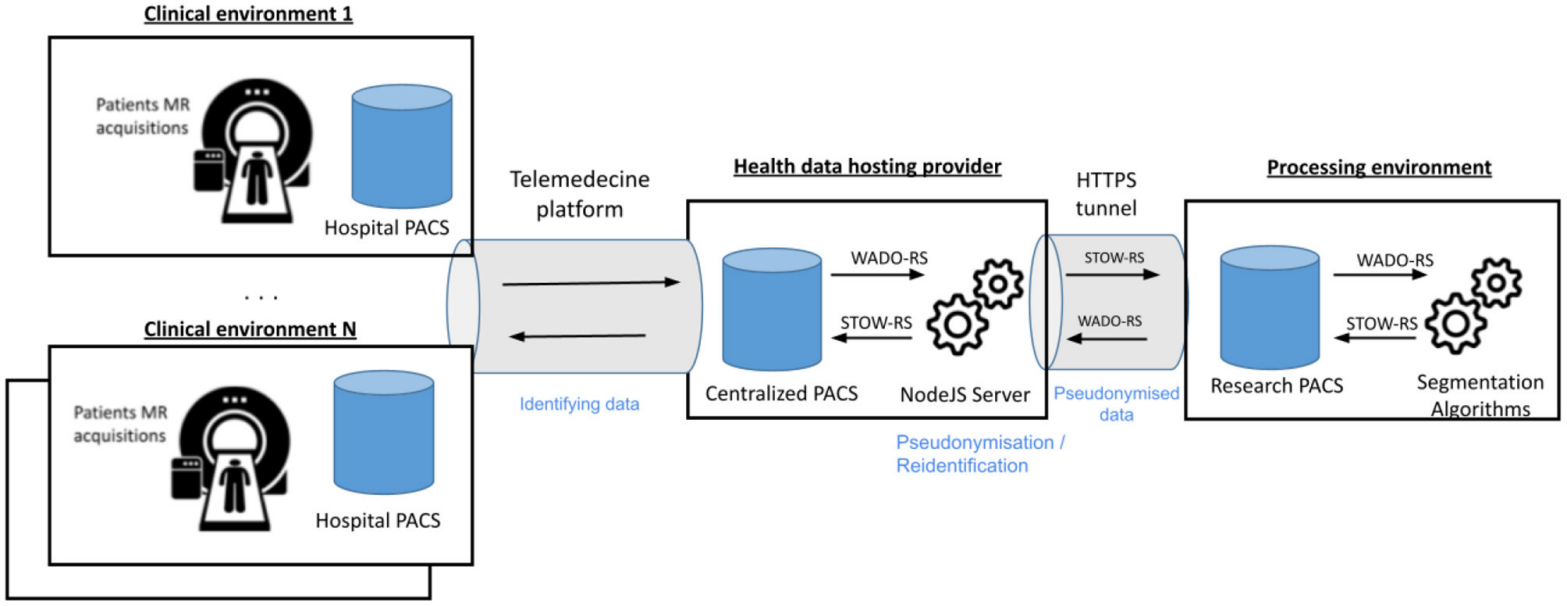
Detailed architecture and interconnections in the MUSIC network. The system is composed of 2 main interconnected PACS (the centralized and the research PACS). The centralized PACS is interconnected to each connected hospital PACS to download the acquisitions to process and to upload the results. The research PACS is dedicated to data processing. The different connections are based on standardized HTTP requests (DICOMweb™) and are secured by an HTTPS tunnel.

In order to process the images outside the hospitals, a set of tools to pseudonymize, securely transfer and reidentify data has been set up. Overall, this module is composed of five main components: the hospital PACS, the centralized PACS, a telemedicine platform, the NodeJS transfer server and the research PACS. These elements and their interconnections are presented in Figure 2. The telemedicine platform is responsible for transferring the medical images from the hospital PACS to the centralized PACS. The latter gathers images coming from different hospitals and is hosted in a certified health data hosting provider. At this stage, patient data are still identified. From here, a clinical research assistant initiates the pseudonymization procedure. An HTTP request is sent to a NodeJS server, also deployed in the same location, with the Universally Unique IDentifier (UUID) of the study to be transferred and the new patient identifier. The server, developed using NodeJS, a JavaScript runtime to develop modular network applications, is a simple server which listens to incoming HTTP requests. It can answer two specific requests: “transfer data” and “import results.” Once it receives a HTTP “transfer data” request, it retrieves the images from the PACS using a DICOMweb™ WADO-RS (Web Access to DICOM Objects Retrieved Study) request, de-identifies the images according to DICOM recommendations (DICOM Supplement 142), and sends the de-identified images to the Research PACS over an HTTPS connection to prevent any attack, using DICOMweb™ STOW-RS (STore Over the Web Retrieved Study). Thanks to this procedure, the pseudonymization is performed inside the health data hosting provider and no identifying data goes out.

To process a set of patient acquisitions, a web application has been developed. This application lists the patients available and is able to run a segmentation over one or multiple selected patients. It also communicates with the research PACS using DICOMweb™, locally downloads the data temporarily and runs the segmentation algorithms from a Python script. Once the images have been processed and segmented, the NodeJS server is notified that results are available by an “import result” HTTP request. It retrieves the images from the research PACS using a DICOMweb™ WADO-RS request, reidentifies the new images using a patented method (ID EP3756123), and stores them in the centralized PACS. The images are finally exported to the radiologist and neurologist to be analyzed via the telemedicine platform. In practice, follow-up data from each patient is thus accessible from any connected clinical environment. The overall transfer time to perform these various tasks is about 15 min per subject (excluding segmentation).

Overall, this workflow has been designed to use only standardized requests for interoperability purposes and can be connected to any telemedicine platform.

#### The Segmentation Module: Detection of New Lesions From Longitudinal Brain MR Images

The visual identification of new lesions in MRI requires the mental processing of a large amount of 3D information and it is common for radiologists to miss notable lesions emerging from one acquisition to another, even for highly-experienced radiologists (7). The segmentation module thus aims at automatically extracting candidate new lesions that will then be highlighted in a dedicated viewer accessible to experts. This design comes with two consequences:

- First, we accept a reasonable amount of false positive candidates that will be naturally considered as irrelevant by image readers, with the counterpart that it increases our chances to detect relevant changes (in other terms, we favor sensitivity over specificity).
- Second, we accept to segment both growing and new lesions without distinction and let the image readers assess the relevance of including each of them into their radiological analysis.

A first natural solution to detection and segmentation of new lesions consists in first, independently segmenting the lesions for each of the two time-points of interest using a dedicated algorithm and second, comparing the resulting segmentation masks (or their associated probability maps) to infer a mask associated to the presence of new lesions. The main advantages of such an approach consist in its ability to stand on the numerous methods developed in the last decades to segment lesions from brain MRI (12) and on the availability of the associated annotated databases. However, by splitting the original problem into two subtasks, this approach disregards the temporal correlation in the images, which may lead to inaccurate segmentations for small lesions or subtle changes. A second fruitful approach thus consists in inferring notable signal changes due to lesions from one acquisition to another directly from the MRI volumes of interest at the two time points, instead of from the two lesion maps (9, 13–18). Such a solution has the advantage of benefiting from all the information available at once and thus maximizing its ability to detect relevant signals of interest. Intuitively, by comparing scans from one session to another, we alleviate the problem from a part of confounding factors due to interindividual anatomical differences. Nonetheless, such a method needs databases of serial MR scans acquired at different time steps and with manually segmented new lesions, which are relatively uncommon as of now.

In this project, we chose to develop a method following this second approach. This method is briefly detailed in the four next subsections. First, we designed a training/testing dataset consisting of a set of pairs of FLAIR, T2-weighted (T2w) and T1-weighted (T1w) acquisitions from 41 MS patients. Second, we set out a pre-processing pipeline so that data of a given patient are appropriately aligned and signal intensity is comparable from one acquisition to another. Third, we trained a deep neural network whose inputs consist, for a given patient, of the two sets of T1w, T2w, and FLAIR images and output consists of the softmax output map associated with the presence of new lesions at each voxel. Fourth, we implemented a few post-processing steps to produce a binary segmentation mask from the network softmax layer. The resulting trained model achieved a true positive rate of 0.83 and an overall rate of false positive of 0.09 on our testing dataset (17 patients, 41 new lesions). Moreover, comparable results were obtained on an additional set of 10 data consisting of acquisitions on Philips and General Electric 3T MRI scanners.

##### Building the Training and Testing Dataset

We designed our segmentation module using a dataset from a previous clinical project (ClinicalTrials ID: NCT02117375) consisting of a set of MR scans from 41 patients acquired on two Siemens 3T MRI scanners (Magnetom Verio, VB17). For each of these patients, data consists of 3D T1w, 2D axial T2w, and 3D FLAIR imaging at two times temporally distant by 1 year. Acquisition parameters were: for 3D T1w: 4 min 30, 1 × 1 × 1 mm, TR = 1,900, TE = 2.26, TI = 900, FA = 9, matrix = 256 × 256, GRAPPA2; for Axial DPw T2w: 2 min 12, 0.7 × 0.7 × 3 mm, TR = 6,530, TE = 9.4/84, FA = 150, matrix = 320 × 320, GRAPPA2; for 3D FLAIR: 5 min, 1 × 1 × 1.1 mm, TR = 5,000, TE = 399, TI = 1,800, matrix = 256 × 256, GRAPPA2.

New lesions from the first acquisition to the second one were manually segmented by an expert and reviewed by another expert using the ITK-SNAP software (http://www.itksnap.org/). This procedure provides the delineation of 152 new lesions. The dataset was split into a learning dataset (24 patients, 111 lesions) and a testing dataset (17 patients, 41 lesions). Data splitting was achieved by stratifying lesions according to their locations (deep white matter, periventricular, juxtacortical, brainstem, cerebellum) and optimizing patients repartition to achieve balanced (60%/40%) training and testing groups with respect to these characteristics.

##### Data Pre-processing

Our preprocessing pipeline, close to the subtraction pipeline proposed in Ref. (16), aims at preparing the T1w, T2w, and FLAIR data so that voxel-wise differences between consecutive scans were as meaningful as possible. Briefly, firstly MR volumes are reoriented in RAS coordinates. Secondly, skulls and skin tissues are removed from the data using a robust registration-based brain extraction method (animaAtlasBasedBrainExtraction, available at anima.irisa.fr, RRID:SCR_017017 and RRID:SCR_01707). Thirdly, baseline and follow-up T1w, T2w, and FLAIR scans are rigidly registered on the FLAIR baseline using a block matching registration method [animaPyramidalBMRegistration (19)]. We used the FLAIR baseline scan as reference for the registration as this is the one generally used by experts in clinical practice. Nevertheless, we did not observe any notable difference in results when modifying the choice of the reference (neither in training, nor in testing). Fourth, images are all cropped using the FLAIR baseline as a mask in order to reduce pointless data. Fifth, bias due to spatial inhomogeneity is estimated using the N4 algorithm (20) and removed from the data (animaN4BiasCorrection). Finally, for each pair of baseline and follow-up images (e.g., FLAIR baseline and FLAIR follow-up) voxel intensities are jointly corrected using a 2 fold procedure: (i) first, their joint histogram is linearly rescaled so that it best fits the y = x line in a least square sense, (ii) second, a Nyul standardization (21) on an in-house multisequence template is applied independently on each acquisition (animaNyulStandardization).

##### Deep Neural Network Architecture and Learning

The core of the segmentation module consists of a fully convolutional neural network that was trained to segment new lesions from a pair of preprocessed FLAIR, T1w, and T2w acquisitions. Specifically, we adopted the nnU-net framework proposed by Isensee et al. [(22), github.com/MIC-DKFZ/nnUNet] that enables training of a 3D U-Net (23) while automating the choice of the hyperparameter values. This framework has been shown to outperform a number of deep learning-based methods on a variety of segmentation tasks. Precisely, our 3D U-Net has 6 input channels (one for each sequence and each time point) of size [160, 192, 64]. To fit this frame, each input image is first resampled to size [0.5, 0.5, 1.1 mm] (median training image resolution) and then each set of 6 images (3 sequences for each of the 2 time points) is split into patches of such a size. Finally, each such 6 × [160, 192, 64] patch is processed independently and aggregated to others to form the final softmax outputs map. Data augmentation included: (i) isotropic rescaling, (ii) 3D rotation, (iii) mirroring in the sagittal plane, (iv) smooth elastic deformations and (v) intensity enhancements and attenuations on lesion voxels (modeling the diversity of signal change due to lesions). This network was trained to minimize the sum of Cross-Entropy and Dice loss over the training dataset and included a drop-out based regularization (with probability = 0.2). Training was performed using a stochastic gradient descent run over 1,000 epochs, each of them consisting of 250 minibatches. Learning was conducted on a GPU NVIDIA Quadro P6000, 24 GB and lasted 10 days. Prediction for a given patient lasted about 6 min (including pre and post-processing) on the same hardware.

##### Data Post-processing

Once the neural network evaluated for a given pair of acquisitions, a binary segmentation map was obtained from the network softmax outputs using the following empirical procedure. First, the softmax outputs map is binarized using a threshold of 0.01. Second, connected components (26-connectivity) were extracted from the resulting binary map. Third, only connected components with volume larger than 12 mm^3^ and including at least one voxel with softmax value >0.1 were selected as new lesions in the final output mask. Last, preprocessed data and the corresponding segmentation mask were resampled to the original baseline FLAIR image slab and resolution.

#### The Visualization Module: Efficient and Adapted Reporting

**Figure 3:**
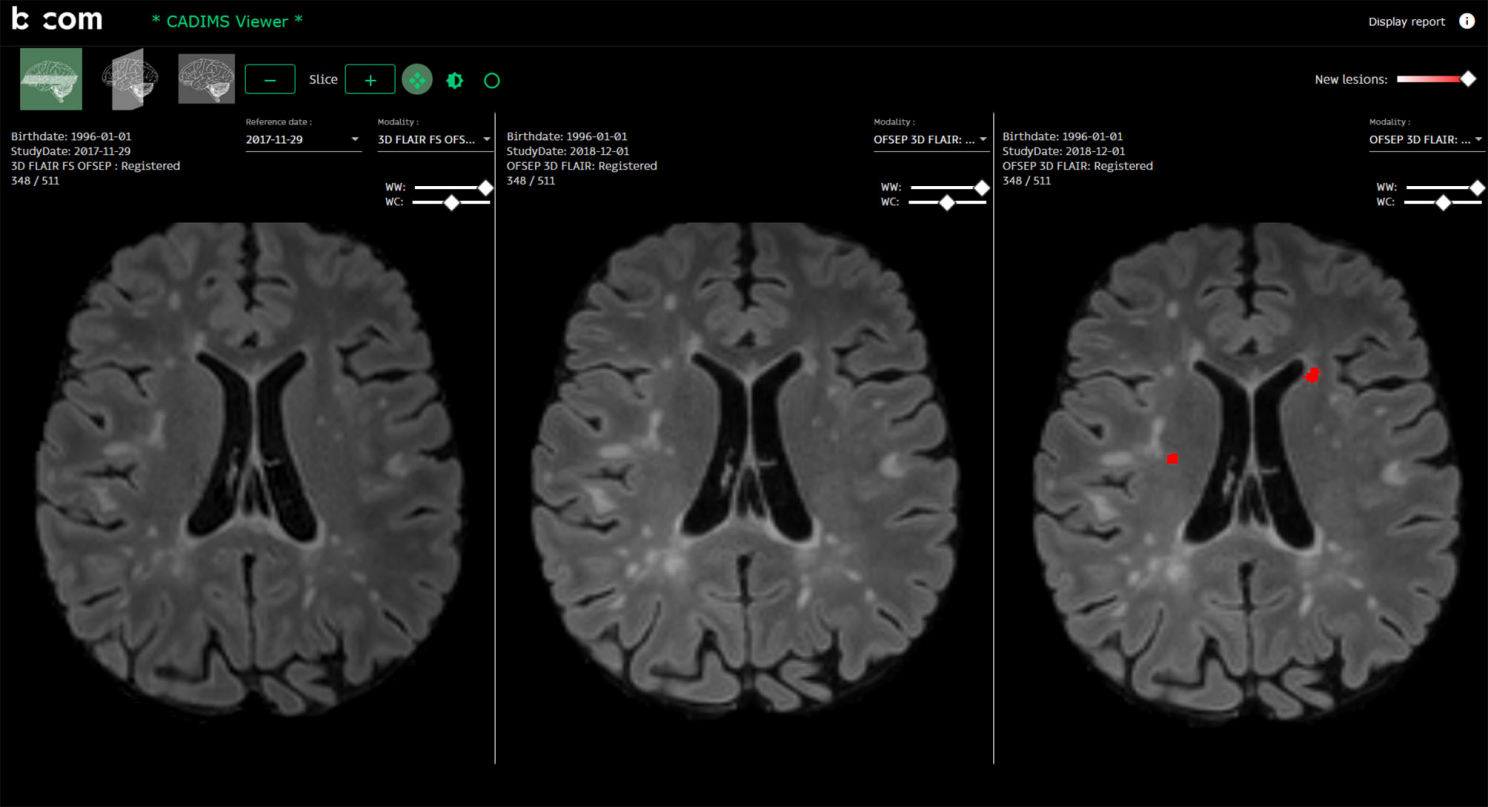
The CADIMS viewer: The CADIMS MRI viewer is usable from a standard web browser. It consists of three synchronized views displaying from left to right (i) the baseline scan, (ii) the follow-up scan and (iii) the follow-up scan with segmented new lesions highlighted in red.

The CADIMS software has been designed to allow a fast and intuitive access to the preprocessed volumes and new lesions segmentation masks (Figure 3, as well as Video available in Supplementary Material). It was built in collaboration with a neurologist and a neuroradiologist following MS patients to meet their clinical needs. It consists of a MRI viewer usable from a standard web browser. It has been developed using the AMI framework (https://github.com/FNNDSC/ami) for the visualization of medical images and integrated in an Angular application. It allows the visualization of DICOM images that are directly retrieved from the hospital PACS using DICOMWeb™, the DICOM Standard for web-based medical imaging. As explained in section The transfer and storage modules: Servers interoperability and data access, the processed scans and the segmentation maps are transferred back via the telemedicine platform and are directly available in the viewer. Moreover, images are still stored durably in the centralized backed up PACS and are also available to any clinicians connected to the MUSIC network.

From a practical perspective, the viewer is composed of three synchronized views where three registered images are visualized simultaneously (from left-to-right: initial image, follow-up image, follow-up image with new lesions mask). The viewer displays the FLAIR images in the axial plane at startup. The other sequences (T1-w, T2-w) and other planes (sagittal, coronal) can be visualized by selecting them on dedicated menus. If more than one follow-up MRIs has been acquired for the same patient, previous acquisitions are also accessible. The viewer also integrates the following basic navigation functionalities: padding, zooming, and intensity windowing, all accessible from the computer-mouse.

### Evaluation of the MUSIC Workflow

In this section, we present the datasets used and the two sets of experiments conducted to assess the added value of the MUSIC workflow on routine clinical practice.

#### Data Sets

Patients from 5 MRI centers were prospectively included in the MUSIC project. All patients were informed and written consents were obtained. All patients were included in the OFSEP (“Observatoire Français de la Sclérose en Plaques”) cohort, registered on clinicaltrials.gov (NCT02889965) and compliant with French data confidentiality regulations. The study was approved by the relevant ethics committee.

Inclusion criteria were chosen to target a population with a substantial number of active patients. They included (i) a diagnosis of MS according to 2017 Mc Donald criteria (2); (ii) a disease duration <10 years; (iii) an Expanded Disability Status Scale (EDSS) score <4; (iv) a follow-up MRI available 10–16 months after the first MRI.

**Figure 4:**
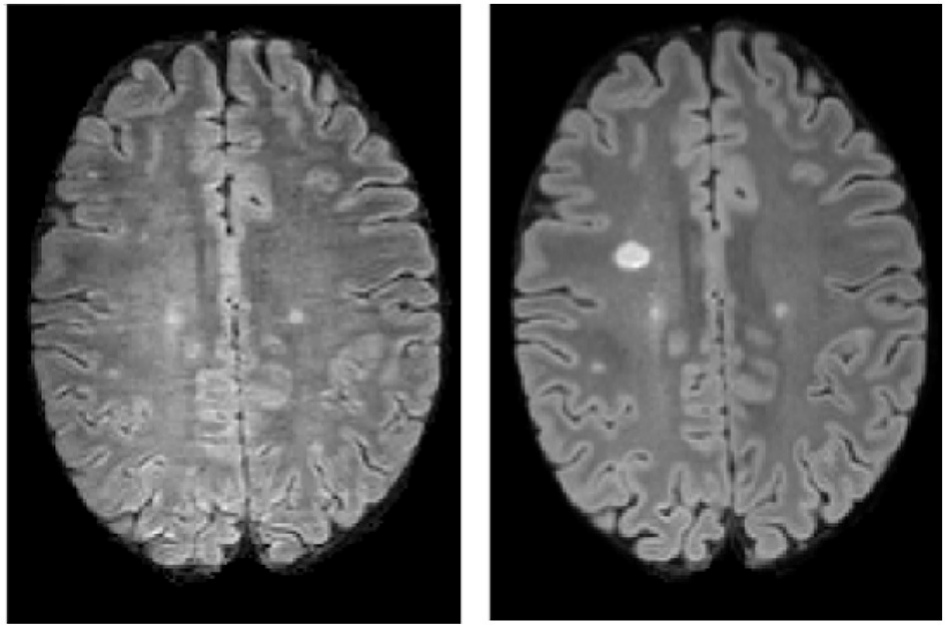
A pair of FLAIR acquisitions from a patient of the evaluation dataset experiencing a low quality baseline scan. **(Left)** Baseline FLAIR axial slice. **(Right)** Follow-up FLAIR axial slice.

Our evaluation dataset consists of 54 pairs (baseline and follow-up) of 2D or 3D FLAIR, T1w, and T2w scans acquired on 9 different 3T MR scanners from Siemens, Philips and General Electrics. Thirty out of the 54 studied MS patients had a follow-up scan on a different MR scanner than the first scan, and 22 out of these 30 on a MR scanner from a different manufacturer. The overall allocation between scanners is depicted in Table 1. All data were acquired according to the OFSEP recommendations (6). The median and range FLAIR, T1w, and T2w spatial resolutions (in mm) were, respectively [1, 1, 1] (range [0.7, 0.7, 0.6]; [1, 1, 1]), [1, 1, 1] (range [0.5, 0.5, 2]; [1, 1, 1]) and [0.7, 0.7, 3] (range [0.5, 0.5, 1]; [1, 1, 3]). Data were not preselected according to quality criteria and a few acquisitions were of lower quality (example in Figure 4). Patients main characteristics were: mean age 35 yo (SD = 10), mean EDSS 1.1 (SD = 1.3), disease duration 3.7 years (SD = 1.3), percent of women = 67%.

**Table 1:** Repartition of patients in the different scanners (each row is a different scanner).

**Manufacturer**	**Version**	**Number of sessions (overall 108)**
Phillips	Ingenia	47
Siemens	Prisma	23
Siemens	Verio	17
Phillips	Ingenia	9
Phillips	Ingenia	5
Siemens	Aera	3
General Electrics	SIGNA Explorer	2
Siemens	Aera	1
General Electrics	SIGNA Explorer	1

#### Experimental Setting

We conducted two experiments involving three experts with different levels of experience: a senior neuroradiologist with 15 years of experience (named “expert 1” below), a senior neurologist with 8 years of experience (named “expert 2” below) and a junior radiologist (named “expert 3” below). Each of these two experiments are detailed below and consisted of the visual analysis of a set of pairs of acquisitions in two different conditions.

##### Impact of the Segmentation Module on Expert Performances

In this first experiment, we assessed the added value of the segmentation module on the ability of each expert to detect new lesions arising between the two time points. This experiment was conducted on 48 patients out of the 54. It consists of a 2-fold procedure. In its first phase, each expert was asked to annotate all notable new lesions—by simply drawing a point near the center of the lesion—from the pre-registered FLAIR, T1w, and T2w volumes for the two time points of interest. Then, in a second phase 2 weeks later, each expert was asked to perform the same exercise with an additional input: the segmentation mask provided by the segmentation module. Annotated lesions as well as time to perform each segmentation were recorded.

This experiment was performed on a dedicated reading system allowing MR volume annotation built from the fsleyes software (https://fsl.fmrib.ox.ac.uk/fsl/fslwiki/FSLeyes). The time measurement was automated. All experts were asked to conduct this experiment in situations as similar as possible to clinical practice. In particular, experts were explicitly instructed not to spend more time reading MRIs than they would have in clinical routine. However, they had to be in a quiet environment so as not to be interrupted during their reading. A few days prior to the first phase, each expert experienced a short session to experiment with the reading system.

##### Impact of the MUSIC Workflow on Routine Clinical Practice

In this second experiment, we explored the added value of the overall MUSIC workflow in clinical practice. This experiment was conducted on 6 patients. Again, it was a 2-fold procedure. In the first phase, each expert was asked to visualize the MRI data and write a radiological report using the fully manual procedure currently in use. Hence, the images were viewed directly from the PACS and MRI for the two time points were manually roughly registered. The presence of new lesions was visually assessed and annotated in the radiological report, without any computer-aided tool. In the second phase of the experiment, 2 weeks later, each expert was asked to repeat the exercise via the MUSIC workflow (i.e., from a user perspective, using the new lesion segmentation mask and realigned data in the dedicated web MRI viewer). The experts measured the time needed to load data, read the MRI and write the report in the two phases. As in the first experiment, experts were explicitly instructed not to spend more time reading MRIs than they would have in clinical routine. They again had to be in a quiet environment so as not to be interrupted during their reading.

#### Statistical Analysis

##### Impact of the Segmentation Module on Expert Performances

First, for each expert and during each phase of this first experiment, detected lesions were colocalized using an automated analysis and manual intervention when necessary. This stage allows us to produce a mapping between each detected lesion, the names of the experts who detected it and the phase (phase 1 or/and phase 2) in which it was detected. Second, each lesion that has been reported, regardless of the phase of the experiment, was labeled as a true positive or a false positive via a consensus reading of all lesions from the two most experienced experts. Finally, we computed:

- The number of lesions detected by each expert as well as the overall number of individual lesions (i.e., counted only once for all experts) detected, for each phase.
- The inter-expert differences on detected lesions within each phase reported as ratio, pairwise Cohen's kappa statistics and multi-rater Fleiss' kappa statistic and associated 95% confidence intervals (CI).
- The number of lesions detected in phase 1 and not in phase 2 and conversely.
- The averaged patient-wise number of lesions detected by experts in each phase, that is compared between phases using a paired student test.
- The number of patients reported with at least one notable lesion by each expert and in each phase, as well as the associated pairwise Cohen's kappa statistics and multi-rater Fleiss' kappa statistic and associated 95% CIs. The overall sensitivity and specificity associated to this categorization (i.e., at least one new lesion vs. no new lesion) was then computed for each phase and tested for equality between phase 1 and phase 2 using a logistic regression including a patient and an expert random effect.
- The pooled inter-expert standard deviation associated to the number of lesions detected in each phase, that is compared between the phases.
- The individual sensitivity together with its 95% CI for each expert and each phase. Moreover, for each expert, sensitivity is tested for equality between phase 1 and 2 using a logistic regression including a patient random effect. Associated odds ratio, *p*-values for odds ratio = 1 and associated 95% CI are reported.

Finally, mean time elapsed for each expert and each phase was estimated and tested for equality between phases using a paired student test.

##### Impact of the MUSIC Workflow on Routine Clinical Practice

First, radiological reports from this second experiment were gathered. Then for each expert and each setting (i.e., using the full MUSIC workflow or using the current manual approach), patients were categorized according to the report as: “no activity,” “1 lesion” or “> 1 lesion.”

Second, the time spent to perform radiological readings for each of the three experts and each of the two settings were summarized and the mean times elapsed in the two settings were tested for equality using a paired *t*-test.

## Results

### Impact of the Segmentation Module on Expert Performances

#### Detection of New Lesions Without the Segmentation Mask

**Figure 5:**
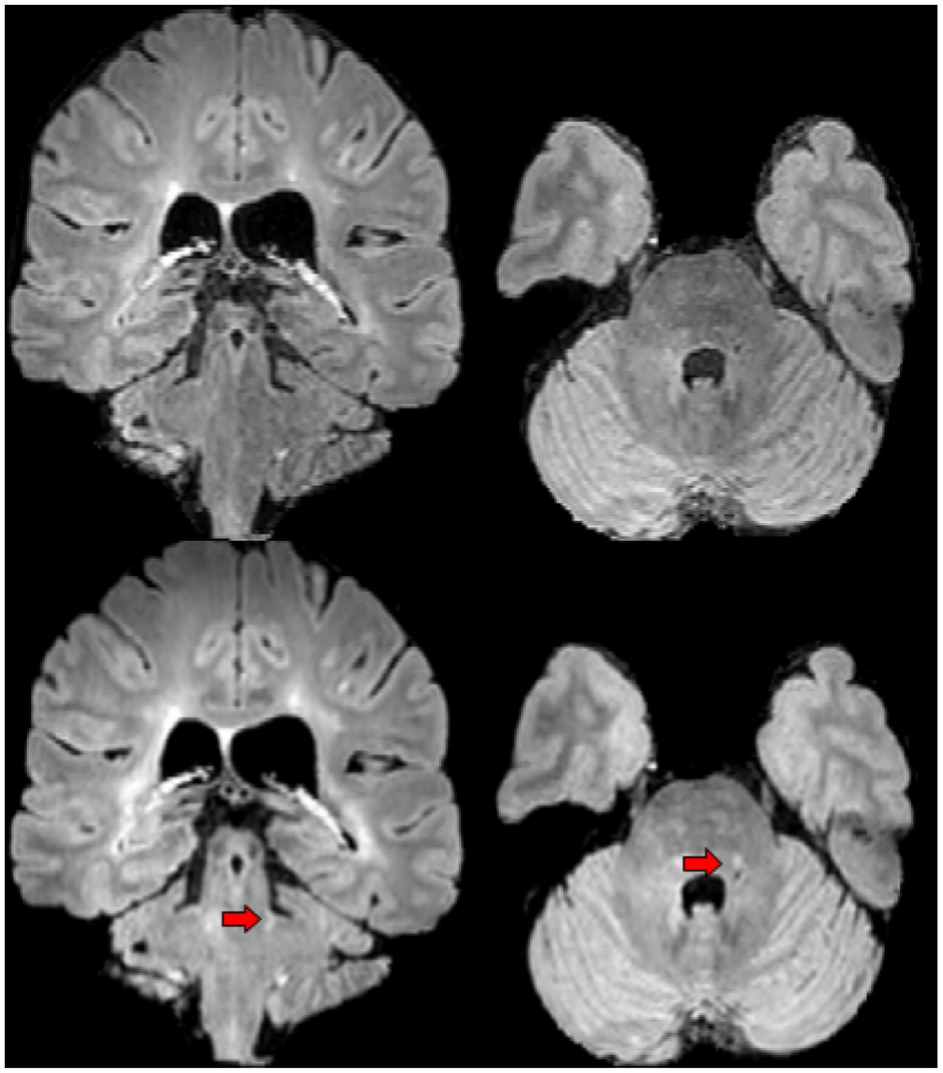
An example of lesion detected by expert 1 in the first phase of the experiment but not by experts 2 and 3. First row shows the baseline FLAIR scan (from left-to-right: coronal and axial view), second row shows the FLAIR scan 1 year later (from left-to-right: coronal and axial view). Red arrows designate the lesion of interest.

During the first phase, overall 113 lesions were detected. The three experts, respectively, detected 90, 83, and 83 new lesions. Table 2 reports the difference of lesions detected from one expert to another as well as the inter-rater Cohen's Kappas, illustrating the high inter-rater variability on detected lesions. Moreover, the overall Fleiss's Kappa coefficient was 0.47 with 95% CI = [0.38, 0.57]. Figure 5 gives an example of a notable lesion detected by only one of the three experts.

**Table 2:** Inter-expert heterogeneity during phase 1.

	**Expert 1**	**Expert 2**	**Expert 3**
Expert 1	–	23/90 κ =0.38 [0.22, 0.53]	17/90 κ = 0.62 [0.48, 0.75]
Expert 2	16/83	–	19/83 κ = 0.44 [0.29, 0.59]
Expert 3	8/83	18/83	–

*The first figures give the number of lesions detected by the expert in row not detected by the expert in column. For example, over its 90 new lesions detected, expert 1 detected 23 lesions that were not detected by expert 2 and 17 that were not detected by expert 3. The second figures (only once per expert combination) give the Cohen's Kappa coefficient and its associated 95% CI*.

At the patient scale, depending on the experts, 19, 19, and 20 patients out of 48 were reported to have at least one sign of MRI disease activity. When combining the different expert segmentations, this number increased to 22. The inter-rater Cohen's Kappa coefficients associated with these patients classifications were: for Expert 1-Expert 2: 0.83 [0.66, 0.99], for Expert 2-Expert 3: 0.78 [0.74, 1], and for Expert 1-Expert 3: 0.96 [0.87, 1]. The overall Fleiss's Kappa coefficient was 0.86 [0.75, 0.97].

#### Detection of New Lesions With the Segmentation Mask

During the second phase (i.e., when segmentation masks provided by the segmentation module were used as supplemental information), the three experts, respectively, detected 114, 111, and 104 lesions. Overall 125 lesions were detected. Table 3 reports the difference of lesions detected from one expert to another in this second phase. The overall Fleiss's Kappa coefficient was 0.59 [0.49, 0.69]. Table 4 details the number of lesions from the segmentation module accepted and rejected by the experts as well as the number of supplemental lesions added. Overall, a large majority of the 121 candidate lesions detected by the segmentation module were accepted by the experts (between 103 and 107 depending on the expert). Eleven lesions out of these 121 were rejected by each of the three experts. After the consensus reading, one supplemental lesion proposed by the segmentation module was rejected, leading to a total of 12 false positive lesions distributed among 8 patients (10% rejection rate) for the segmentation module. At the patient scale, depending on the experts, 24, 23, and 23 patients were reported to have at least one sign of disease activity. When combining the different expert segmentations, this number rises to 25. The inter-rater Cohen's Kappa coefficients associated with these patients classifications were: for Expert 1-Expert 2: 0.96 [0.88, 1], for Expert 2-Expert 3: 0.92 [0.80, 1], and for Expert 1-Expert 3: 0.88 [0.74, 1]. The overall Fleiss's Kappa coefficient was 0.92 [0.83, 1].

**Table 3:** Inter-expert disparity during phase 2.

	**Expert 1**	**Expert 2**	**Expert 3**
Expert 1	–	10/114 κ = 0.44 [0.29, 0.59]	16/114 κ = 0.51 [0.34, 0.69]
Expert 2	7/111	–	11/111 κ = 0.69 [0.53, 0.83]
Expert 3	6/104	4/104	–

*The first figures give the number of lesions detected by the expert in row not detected by the expert in column. For example, over its 114 detected new lesions, expert 1 detected 10 lesions that were not detected by expert 2 and 16 that were not detected by expert 3. The second figures (only once per expert combination) give the Cohen's Kappa coefficient and its associated 95% CI*.

**Table 4:** Relevance of the segmentation masks produced by the segmentation module.

	**Expert 1**	**Expert 2**	**Expert 3**
Accepted lesions	105	107	103
Rejected lesions	16	14	18
Supplemental lesions	9	4	1

*Number of lesions accepted, rejected and added by the different experts when using the segmentation mask as supplemental information*.

#### Consensus Lesions Reading and Patient Characteristics

Overall, 138 individual lesions were reported by the experts during the two phases. Two of these 138 lesions were then discarded during the concerted reading (one was reported in phase 1 and the other one in phase 2). The patient-wise repartition of lesions is given in Supplementary Figure 1. Briefly, the median lesion number was 1, ranging from 0 to 18. Twenty-two patients (about 46%) did not develop new lesions.

#### Comparison of New Lesions Detection With and Without the Segmentation Mask at the Lesion Scale

**Figure 6:**
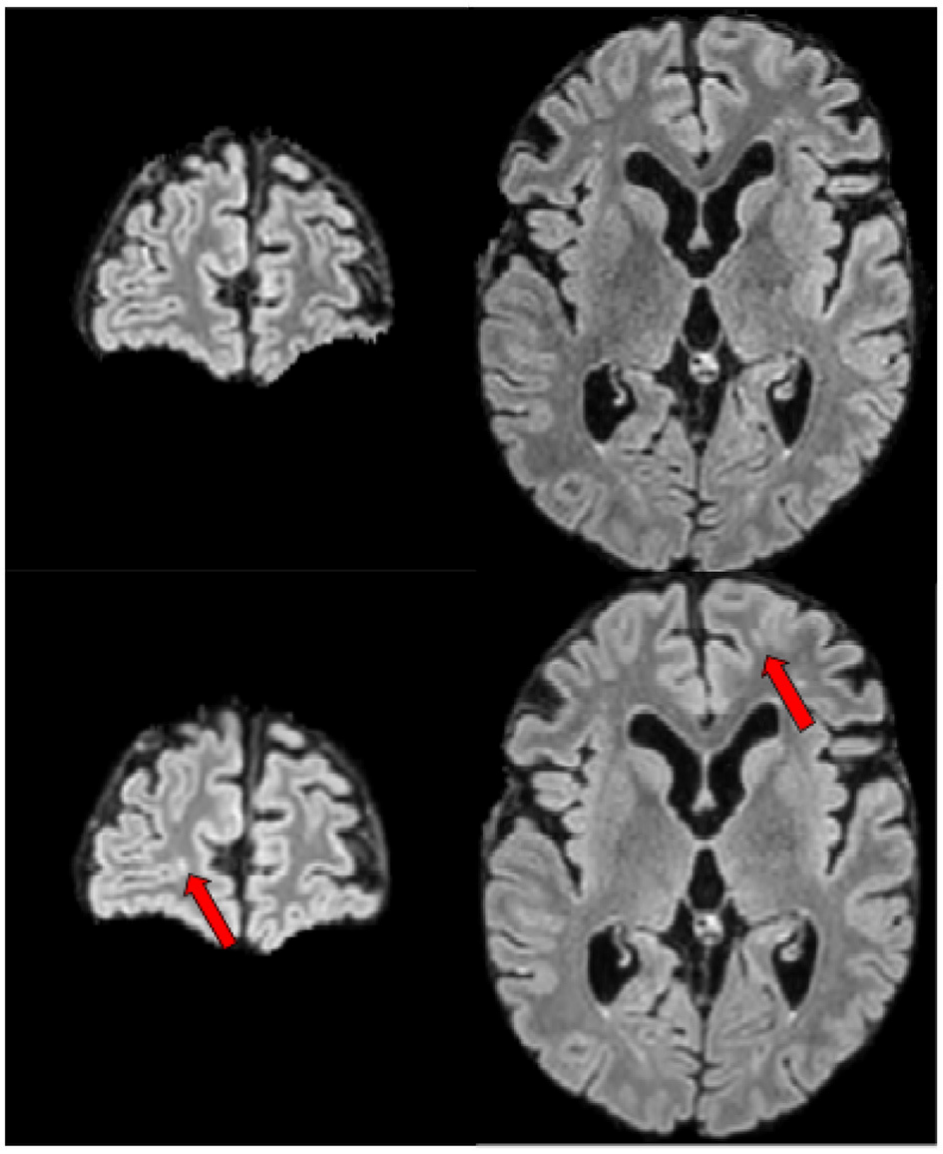
Example of a lesion detected by none of the experts in the first phase of the experiment, detected by the segmentation module and accepted by all experts in the second phase of the experiment. First row shows the baseline FLAIR scan (from left-to-right: coronal and axial view), second row shows the FLAIR scan 1 year later (from left-to-right: coronal and axial view). Red arrows designate the lesion of interest.

By comparing lesions detected in the two phases (and excluding the two false positive lesions), we identified 103 cases of lesions that were not detected by an expert in the first phase but were detected by this expert in the second phase. Figure 6 displays an example of lesion that was detected by the segmentation module and accepted by the three experts in the second phase of the experiment but that was reported by none of the three experts during the first phase of the experiment. Conversely, we identified only 30 cases of lesions that were first detected by an expert in the first phase but not detected in the second phase.

Table 5 reports the statistics on lesion detection averaged over patients and highlights the added value of the segmentation module to increase expert performance. Similarly, Table 6 reports increased ability of each expert to detect new lesions using the segmentation module. Finally, Table 7 reports the statistics on elapsed time for each of two phases for the three experts and highlights the gain in expert processing time brought by the use of the segmentation mask.

**Table 5:** Statistics on lesion detections averaged over patients and differences between phase 1 (lesions detection only using patient acquisitions) and phase 2 (lesions detection using patient acquisitions and segmentation mask produced by the segmentation module).

	**Phase 1**	**Phase 2**	**Phase 2 to Phase 1 differences value, [95%CI] and *p*-value for no difference**
Mean number of detected lesion per patient and per expert	1.8 Lesion	2.3 Lesion	Mean difference = 0.5 [−0.78, −0.23] *p* = 5.10^−4^
Pooled standard deviation from interexpert variability	0.76 Lesion	0.55 Lesion	Mean difference = 0.09 [−0.02, 0.29] *p* = 0.12

*First row: averaged patient-wise number of lesions detected in phase 1 (column 1) and 2 (column 2) and mean difference, associated 95% CI and p-value for a null difference between the two phases (column 3). Second row: pooled inter-expert standard deviation associated with lesions number detected in phase 1 (column 1) and 2 (column 2) and mean difference, associated 95% CI and p-value for a null difference between the two phases (column 3)*.

**Table 6:** Ability of each expert to detect a new lesion during the two phases.

	**Phase 1 sensitivity** **[95%CI]**	**Phase 2 sensitivity** **[95%CI]**	**Phase 2 to Phase 1 differences odds ratio, [95%CI] and *p*-value**
Expert 1	0.66 [0.58, 0.74]	0.84 [0.76, 0.90]	2.77 [1.55, 5.15] *p* = 7.10^−4^
Expert 2	0.60 [0.51, 0.68]	0.82 [0.74, 0.88]	3.35 [1.84, 6.29] *p* = 8.10^−5^
Expert 3	0.61 [0.52, 0.69]	0.75 [0.68, 0.83]	2.31 [1.33, 4.10] *p* = 3.10^−3^

*For each expert (in row): the ratio of new lesions detected over the overall 136 lesions (the overall number of lesions detected on all patients, by all experts during the two phases and confirmed during the concerted reading) as well its 95% CI for phase 1 (first column) and phase 2 (second column) and difference between phase 1 and phase 2 (third column)*.

**Table 7:** Statistics on time elapsed for each of two phases for the three expert and comparison between the two phases.

	**Phase 1 duration** **(mean, [range])**	**Phase 2 duration** **(mean, [range])**	**Phase 1 to Phase 2 difference** **[mean, (sd), *p*-value]**
Expert 1	317s [144, 807]	232 s [91, 603]	85 s (137 s) *p* =10^−5^
Expert 2	283s [125, 847]	204s [93, 511]	78 s (126 s) *p* = 10^−5^
Expert 3	272 s [146, 525]	160 s [82, 287]	112 s (79 s) *p* = 10^−13^

*First column: Mean time and associated range associated with the processing of the 48 patients in Phase 1. Second column: same elements for phase 2. Third column: Mean time reduction from Phase 2 to Phase 1, associated standard deviation and p-value for a null time reduction*.

#### Comparison of New Lesions Detection With and Without the Segmentation Mask at the Patient Scale

Table 8 provides a contingency table summarizing the numbers of patients that were identified as having no lesion, one lesion or more than one lesion during the two phases of the experiment. Moreover, 20 patients (by adding those identified by each expert) were wrongly identified as having no new lesion in the first phase, against only 8 patients in the second phase of the experiment. The overall sensitivity at the patient scale (i.e., no new lesion vs. at least one new lesion) was 0.74 in the first phase, and 0.90 in the second phase of the experiment (*p*-value for unit odds ratio = 0.003). Moreover, for each expert and each phase, the patient-wise specificity was equal to 1.

**Table 8:** Contingency table of numbers of patients reported with no (0), one (1), or more than one (>1) lesion in the two phases of the experiment.

		**Phase 2**		
		**0**	**1**	**>1**
Phase 1	0	71 (23, 23, 25)	13 (5, 5, 3)	2 (1, 1, 0)
	1	3 (1, 2, 0)	10 (3, 3, 4)	2 (0, 0, 2)
	>1	0 (0, 0, 0)	2 (1, 0, 1)	41 (14, 14, 13)

*In each cell, the top figure indicates the overall number of reported patients while the three bottom figures give, respectively, these figures for expert 1, expert 2, and expert 3*.

When assessing the reported new lesions as detected by the segmentation module (i.e., with no adjustment by an expert), we computed a sensitivity of 0.90 and specificity of 0.84 at the patient scale.

### Impact of the MUSIC Workflow on Routine Clinical Practice

Supplementary Table 1 gives the main elements of reporting for each expert and each patient when using a standard manual examination of data from the clinical PACS (phase 1) and when using the MUSIC workflow (phase 2). In particular, expert 2 and expert 3 reported two patients without activity in phase 1 (patients 2 and 3) while they reported a notable new lesion for these same patients in the second phase. Finally, Table 9 gives the mean time elapsed by the three experts in the two settings. Mean times elapsed in the two settings differ significantly [mean difference = 2′45″ (SD = 2′00″), *p* = 10^−4^].

**Table 9:** Mean (standard deviation) time elapsed to perform radiological readings of the patients of interest for each of the three experts and each of two phases of the second experiment.

	**Expert 1** **mean** **(standard deviation)**	**Expert 2** **mean** **(standard deviation)**	**Expert 3** **mean** **(standard deviation)**
Phase 1	4′45″ (1′30″)	7′00″ (3′00)	5′15″ (1′30″)
Phase 2	3′15″ (0′30″)	3′00″ (0′30″)	3′45″ (0′30″)

## Discussion

While there is a growing number of methodological works addressing the question of automating the detection of new MS lesions from one acquisition to another using deep learning techniques [e.g., (7, 9, 10)], the integration of such tools in clinical practice as an aid to clinicians and the associated added-value on the resulting radiological reports have not been fully evaluated. This work aims at providing elements to document these two points. In particular, we described a fully-integrated workflow and showed that the proposed workflow increases MRI reader performance to detect new MS lesions on longitudinal MRI scans while decreasing MRI comparison time. Beyond the number of lesions detected, our workflow has an impact on the number of MS patients classified as stable or active based on their MRI, even by the most experienced neuroradiologist. It may therefore have substantial consequences on the therapeutic management of MS patients.

### Visual Detection of New MS Lesions Is a Complex Task

First, as previously reported, we observed a high inter-expert variability in the detection of new FLAIR lesions (24, 25). In practice, a significant part of this variability is not due to differences of MR signal interpretations but related to the difficulty to visually notice them within the whole 3D volumes of interest. Indeed, while we did not investigate the intra-expert variability, a previous study reported a mean intraobserver kappa score for new lesions detection at 0.72 (25). As expected, in the present study, the expert with the highest level of experience (neuroradiologist with 15 years of experience) detected a higher number of new lesions than the other clinicians.

### Automated New Lesion Segmentation Tools Provide a Relevant and Valuable Aid for Clinicians

Second, we observed that the use of lesion masks produced by the lesion detection module significantly increases the number of lesions detected regardless of the level of expert's experience (more than 15% more lesions with the MUSIC workflow than without). This observation is in line with recent studies not involving deep learning based segmentation (26–28). In parallel, while not significant, we also observed a natural reduction of the inter-expert variability when using the segmentation masks.

It is also interesting to note that we deliberately put ourselves in difficult conditions by including longitudinal data acquired on different scanners in 56% of the cases. These conditions are representative of the follow-up conditions in clinical practice where patients may be followed in different centers and on different scanners. Moreover, we did not discard lower quality acquisitions from the study. Despite these heterogeneities the segmentation module provides valuable aid to clinicians. In particular, we did not observe evidence of mean differences in sensitivity of the segmentation module depending on whether baseline and follow-up data come from the same scanner or from two different scanners/brands (mean difference = 0.10, *p* = 0.44 for “same scanner vs. different scanners,” mean difference = 0.04, *p* = 0.77 for “same brand vs. different brands”). This point must however be mitigated by our sample size that may be too low to evidence subtle mean differences. Meanwhile, the rejection rate, i.e., the percentage of candidate lesions detected by the segmentation module that were rejected by the experts was moderate (about 10%) and most segmentation masks (about 80%) did not present any false positive lesions. Overall, this rate must be considered in light of our methodological choices. Indeed, in this work, we chose to accept the presence of a reasonable amount of false positives (favoring sensitivity over specificity). Optimizing the balance between the number of false negative lesions (increasing experts' acceptance and comfort) and the number of true positive lesions (decreasing the probability to miss a new lesion) may consist of interesting future directions. In particular the segmentation module, and especially the post-processing rules that drive most of this balance, could be modified for this purpose. This optimization could also depend on acquisition characteristics (e.g., acquisition signal-to-noise ratio, scanner brand) to reduce potential effect of these factors on performance.

It is worth noting that we do not think these results are intrinsically related to our segmentation module. Indeed, while being built on state-of-the-art solutions and exhibiting satisfying performances, it may be replaced by other recent methods of the literature [e.g., (7–10)]. Our aim is not to show the superiority of our segmentation module but to evidence the potential impact of using state-of-the-art segmentation methods on MS clinical practice.

### Using a New Lesion Segmentation Mask Was Well-Received by the Experts

Importantly, all three experts reported a satisfying and comfortable reading experience when using the segmentation mask as an aid, especially with the full workflow (Experiment 2). Additionally, for each of them, the time spent to analyze the images was significantly reduced in the second phase of the experiments.

More specifically, the three experts were satisfied by the information provided by the segmentation masks and reported that the segmentation module offered very good performances. While this result is satisfactory, it also raises issues related to the confidence to place in these segmentation masks, especially regarding their potential lack of sensitivity. As an example, in the second phase of our first experiment, expert 3 only added 1 supplemental lesion to those proposed by the segmentation module, while being the expert exhibiting the highest gain of processing time between Phase 1 and Phase 2. While on average, the performances of expert 3 were notably superior with the MUSIC workflow than without, this observation also suggests a risk for the experts to place too much trust into the automated outputs. We think that ways to mitigate such risk, such as confidence intervals or uncertainty estimation (10, 29), have to be considered in future methodological developments.

### Automated New Lesion Segmentation Tools May Have Substantial Consequences on the Therapeutic Management of MS Patients

Beyond lesion-wise statistics, our results suggest that the use of segmentation masks has also consequences at the patient level. Indeed, in the first experiment, it allows each expert to identify three to five supplementary active MS patients (i.e., with at least one new lesion on MRI). This result, which may seem important, should be interpreted in relation to our dataset, including particularly active patients. This high activity rate is well-explained by our inclusion criteria, selecting patients at the early stage of the disease.

Moreover, it would be interesting to evaluate the consequences on patient management by the clinician (in particular with respect to potential treatment changes). Indeed, the appearance of new lesions under treatment is recognized as being prognostic of an increased risk of clinical relapse and of disability progression. It consequently often leads to a change of treatment in clinical practice (30, 31). This point could be evaluated in a future study including the neurologists in charge of these patients. In the longer term, the objectives would be to evaluate the impact of such a tool on the evolution of disability in patients and on the costs of managing the disease. Finally, it would be interesting to evaluate this workflow from the patient's point of view. There is indeed a potential added-value of a straightforward visualization enhancing new lesions to facilitate the clinician-patient dialogue, especially to argue for a change of DMT.

### Limits and Perspectives

Our study has several limitations that need to be discussed. First, our evaluation must be interpreted in light of our population, which exhibits a high prevalence of new lesions due to our inclusion criteria. Indeed, we voluntarily put ourselves in a setting where the inter-expert and intra-expert variabilities are exacerbated and, as a consequence, where a computer-guided aid is likely to offer a high added-value. If the number of active patients had been lower, we can reasonably assume that average expert performances without the computer-guided aid would have been better and that the resulting added-value of our workflow would have been less pronounced.

Secondly, all FLAIR, T2-w, and T1-w images were used as input to the automatic lesion detection module. These 3 sequences correspond to those currently recommended in the OFSEP protocol in France (6) and are mostly performed in clinical routine for the follow-up of MS patients. However, in some cases, due to time constraints, some of these sequences are not acquired. Our segmentation module therefore needs to be adapted and evaluated to deal with this configuration.

Thirdly, while it is consistent with that used in other studies (26, 32), our evaluation sample size (54 patients) is limited. It will be interesting to evaluate our workflow and confirm our results on a larger sample from all centers involved in the follow-up of MS patients in our region. Moreover, some MRI scanners are under-represented in our sample (as GE scanners) and the size of our cohort did not allow us to analyze the performance of our tool by subgroup, e.g., according to the type of MRI scanners used. Despite these limitations, overall, the added value of our segmentation module compared to a standard radiological reading appears clearly significant, both on the number of lesions detected and on the time to perform this task.

Fourth, the fact that all readings were firstly performed without assistance (phase 1) and secondly using the segmentation mask as an aid (phase 2) may have introduced a bias that would have been reduced by using a dedicated design. However, we are confident about the lack of such substantial bias. Indeed, a 2-week period was included between the two phases and this period consisted, for each expert, of a dense clinical and radiological activity. Moreover, the number of data analyzed was consequent and the order of analysis of the patients was different between the two readings.

Fifth, our segmentation module could be improved following recent methodological advances. In particular, a two-path encoder that extracts hierarchical features for each time-point separately, while allowing for an exchange of information at certain levels of abstraction, might be explored in the future (33). In parallel, the design of methods using both a joint analysis of the baseline and follow-up acquisitions (as in the present work) and an analysis of each cross sectional segmentation probability maps, obtained from dedicated algorithms, could maximize the use of the information available in the different annotated databases (34, 35). In particular, these latter segmentation probability maps could be obtained estimating the confidence maps associated with the presence of lesions (10), that have already shown their interests to detect new MS lesions.

Sixth, our experiments were limited to follow-up with two time points and did not include settings with more time points. Our workflow can actually deal with such settings by processing the data sequentially, using the first baseline images as reference target for registration, and performing segmentations independently for each consecutive pair of acquisition sessions.

Finally, in our study, we mainly evaluated our workflow at the lesion scale. Evaluating the impact of such workflow at the patient scale, and in particular its consequences on patient's management (continuation or change of treatment, effect on disability progression for example) is a final objective that we did not fully address in this study and constitutes the future directions of our work.

## Conclusion

The workflow proposed in this paper consists of a fully-integrated and user-friendly computer-aided MRI reading system, potentially accessible to all neurologists and radiologists in a given area. Importantly, the aid provided by the segmentation module significantly improved both the number of new FLAIR lesions detected by MRI-readers, including highly experienced ones, the number of patients classified as having active disease, and the time spent interpreting follow-up MRIs. These results should make us think about how to widely disseminate such workflows, to allow an optimized follow-up for all MS patients wherever they are followed and whatever the level of expertise of their clinicians.

## Data Availability Statement

The original contributions presented in the study are included in the article/Supplementary Material, further inquiries can be directed to the corresponding author/s.

## Ethics Statement

The studies involving human participants were reviewed and approved by CPP Ille-de-France VI (POCADIMS protocol). The patients/participants provided their written informed consent to participate in this study.

## Author Contributions

BC, AK, GP, FG, J-CF, and EC: writing paper. AK, GE, J-CF, OC, PL'H, EC, and GE: designing framework. BC, GP, BL, FG, OC, and EC: designing framework modules. BC, AK, J-CF, GP, and GE: designing experiments. BC and AK: analysis experiments. AK, J-CF, NE, and RC: expert segmentation. All authors contributed to the article and approved the submitted version.

## Funding

This study received funding from Biogen, Teva Santé, Novartis Pharma, Roche SAS, Merck Serono, Sanofi. The funders were not involved in the study design, collection, analysis, interpretation of data, the writing of this article or the decision to submit it for publication. As a part of the MUSIC project, the academic POCADIMS study (NCT03205280) was funded by the Programme Hospitalier de Recherche Clinique-Interregional (PHRC-I) 2016, Groupement Interrégional de Recherche Clinique et d'Innovation Grand Ouest. The study was supported by a grant from the Institut des Neurosciences Cliniques de Rennes (INCR).

## Conflict of Interest

GP and EC were employed by b.com. PL'H was employed by Biotrial. The remaining authors declare that the research was conducted in the absence of any commercial or financial relationships that could be construed as a potential conflict of interest. The authors declare that this study received funding from Biogen, Teva Santé, Novartis Pharma, Roche SAS, Merck Serono and Sanofi. The funders were not involved in the study design, collection, analysis, interpretation of data, the writing of this article or the decision to submit it for publication.

## Publisher's Note

All claims expressed in this article are solely those of the authors and do not necessarily represent those of their affiliated organizations, or those of the publisher, the editors and the reviewers. Any product that may be evaluated in this article, or claim that may be made by its manufacturer, is not guaranteed or endorsed by the publisher.

## Supplementary Material

Supplementary Figure 1 Number of patients for each new lesion count (from 0 to 18).

Supplementary Video The CADIMS viewer in action: an example of data visualization in the CADIMS viewer.
