# Supplementary figures and images for: A Clinically-Compatible Workflow for Computer-Aided Assessment of Brain Disease Activity in Multiple Sclerosis Patients
Source: Front Med (Lausanne). 2021 Nov 3;8:740248. doi: 10.3389/fmed.2021.740248 (PMC8595265; doi:10.3389/fmed.2021.740248)

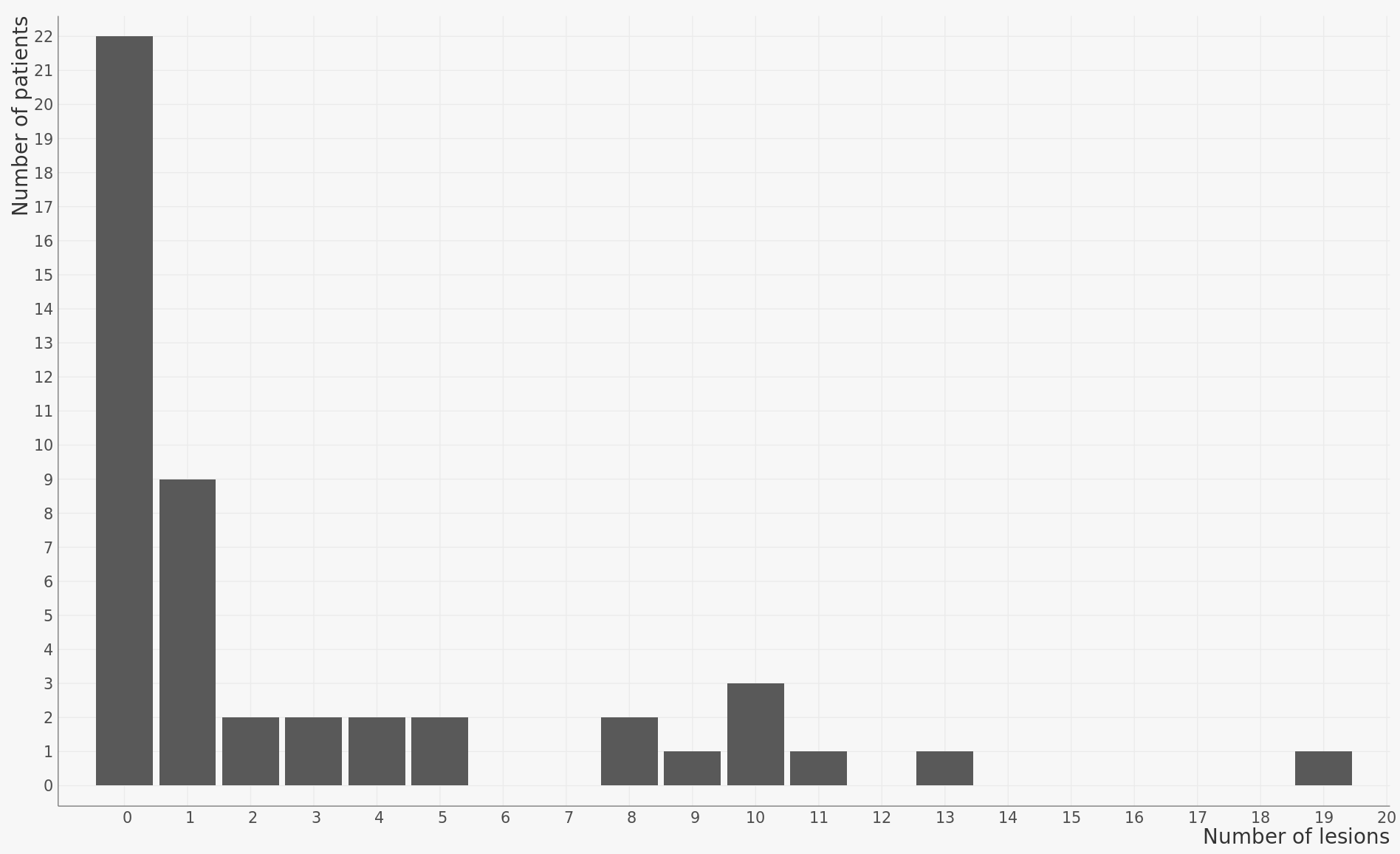

Supplement: Supplementary Figure 1 — Number of patients for each new lesion count (from 0 to 18). [file Image_1.PNG]
