# Supplementary material for: A Clinically-Compatible Workflow for Computer-Aided Assessment of Brain Disease Activity in Multiple Sclerosis Patients
Source: Front Med (Lausanne). 2021 Nov 3;8:740248. doi: 10.3389/fmed.2021.740248 (PMC8595265; doi:10.3389/fmed.2021.740248)
Supplement: Supplementary file 3 [file Table_1.DOCX]

|  | **Expert 1** | | **Expert 2** | | **Expert 3** | |
| --- | --- | --- | --- | --- | --- | --- |
|  | Standard approach | Music  workflow | Standard approach | Music  workflow | Standard approach | Music  workflow |
| **Patient 1** | no activity | no activity | no activity | no activity | no activity | no activity |
| **Patient 2** | 1 lesion | 1 lesion | no activity | 1 lesion | no activity | 1 lesion |
| **Patient 3** | 1 lesion | 1 lesion | no activity | 1 lesion | no activity | 1 lesion |
| **Patient 4** | 1 lesion | > 1 lesion | 1 lesion | > 1 lesion | > 1 lesion | > 1 lesion |
| **Patient 5** | no activity | no activity | no activity | no activity | no activity | no activity |
| **Patient 6** | > 1 lesion | > 1 lesion | >1 lesion | >1 lesion | > 1 lesion | > 1 lesion |
| Supplemental Table 1**: Main elements from radiological reports of the 6 patients performed using the currently used approach (standard approach) and the full MUSIC workflow.** For each patient, we summarize radiological reports according to three labels: “no activity”, “one new lesion” or “1 > lesion”. | | | | | | |

## 
